# Supplementary material for: Energy-efficient UAV communication: A NOMA scheme with resource allocation and trajectory optimization
Source: PLoS One. 2024 Apr 16;19(4):e0301819. doi: 10.1371/journal.pone.0301819 (PMC11020866; doi:10.1371/journal.pone.0301819)
Supplement: Attachment — Submitted filename: Response to Reviewers.docx [file pone.0301819.s002.docx]

Dear Editor and Reviewers:

On behalf of my co-authors, we are very grateful to you for giving us the last opportunity to revise our manuscript. We appreciate you very much for your positive and constructive comments and suggestions on our manuscript entitled “A NOMA Scheme with Resource Allocation and Trajectory Optimization” (PONE-D-23-21957)

We have studied the comments of all Reviewers carefully and tried our best to revise our manuscript according to the comments. The following are the responses and revisions. We have made in response to the questions and suggestions on an item-by-item basis. Thanks again to the hard work of the editor and reviewers.

**Reviewer #1:**

***Comment 1:*** *Replace “un-manned aerial vehicle (UAV)” with “unmanned aerial vehicle (UAV)” in Abstract.*

***Response***: Thank you for your valuable comment, we have revised the description carefully.

***Comment 2:*** *Replace “the target optimization value is fast convergence” with “the target optimization value converges fast”.*

**Response:** Thank you for your valuable comment, we have revised the sentence carefully.

***Comment 3:*** *From the statement “is superior to other benchmarks.”. What benchmarks are the authors referring to? Please state them. Furthermore, it is important for the authors to clearly state the improvement values (as a percentage) of the proposed schemes over the baselines.*

**Response**: We are really sorry that we did not explain the benchmark. The benchmark in simulation has been emphasized with OMA, moreover, the improvement values as a percentage has been added in more than 99.6% in Fig 5 our revised manuscript.

***Comment 4:*** *Why choose the exact figure of 300m? Is it about 300m? And the authors should state why such performance in the abstract. In general, the abstract can be better presented.*

**Response**: Thank you for your valuable comment. As shown in Fig 7a, the EE curve of the three user clustering algorithms are coincided gradually after 300m, and the value of EE no longer has a significant gap with the change of height. And according to equation (1), as the increasing of 3D distance between the ground users and the UAV, the difference of channel gain of all users was not obvious, however, the difference of channel gain has much influence on performance of NOMA (power domain). The effect of the user clustering algorithms isn’t obvious after the height of 300m for the UAV. Therefore, 300m was chosen in our manuscript.

***Comment 5:*** *On page 2, replace “in rescue fleetly when disasters occurring” to “**in rescue fleet when disaster occurs”*.

**Response**: We are really sorry for the grammatical errors, similar errors have been checked in our revised manuscript carefully.

***Comment 6:*** *The authors should incorporate closely related works that deploy UAVs in similar scenarios. The authors ignored works that have applied other approaches to address these problems such as interference management and energy efficiency (EE). Therefore, I suggest to the authors consider the following references since they were compared against cluster-based approaches presented in this work:*

*[A] Communication-Enabled Deep Reinforcement Learning to Optimise Energy-Efficiency in UAV-Assisted Networks, Vehicular Communications, Volume 43.*

*[B] Multi-Agent Deep Reinforcement Learning For Optimising Energy Efficiency of Fixed-Wing UAV Cellular Access Points," ICC 2022 – IEEE International Conference on Communications, 2022, pp. 1-6.*

**Response:** Thank you for your valuable comment, the manuscript has been carefully revised with red font in the section of related work.

***Comment 7:*** *From the related work section, it’s unclear what the key contribution of the work is. The authors should incorporate a table that highlights each of the closely related work and include those in [A] and [B].*

**Response：**Thank you for your valuable comment. Table 1 shows a summary of related works in our revised manuscript.

***Comment 8:*** *The authors will do well to provide the control overhead of the proposed approach with respect to the baselines used in the evaluation.*

**Response：**Thank you for your valuable comment, the work has been amended. The performance of the proposed algorithms are improved by more than 99.6%, 104%, and 111%, respectively.

***Comment 9:*** *Lots of mathematical notations used. Please provide a list of notation table.*

**Response:** Thank you for your valuable comment, Table 2 was provided in our revised manuscript.

***Comment 10:*** *We understand that the ground users may be mobile, however, from the system model, the authors consider the deployment of static ground users. How does you algorithm account for the possible change of position of the ground users? Please provide justification for this.*

**Response:** Sorry for our unclear expression. The location of groud users in our paper is immobile. In this paper, we consider that the location of user is fixed, at the same time, UAV was regarded as mobile aerial base station serving ground users.

***Comment 11:*** *How did the authors arrive at the chosen altitude for the fixed-winged UAVs? Authors may provide references to backup their chosen parameters. Ref [B] may be of help.*

**Response:** After the 2D horizontal position of the UAV is obtained, it is considered that the flight process of UAV’s height rising with the increase of flight time slot t in uniform motion according to the kinematic and dynamic characteristics of fixed wing. Set the height increment for each flight to 50m.

***Comment 12:*** *The overall language can be further improved, with proper use of words.*

**Response:** Thank you for your valuable comment. The manuscript has been carefully revised with red font.

***Comment 13:*** *The authors have adopted cluster-based approaches, however, it is unclear who* *performs the clustering task. Moreover, the knowledge of the position of all ground users within the coverage area must be known beforehand. This assumption may not be realistic. The authors should provide a justification for this.*

**Response:** UAV performs the clustering task. With the rapid development of network navigation systems and smart devices such as mobile phones, the position of ground users can be obtained according to the existing satellite signals and systems, for example, in the actual process of travel, people can obtain the road ahead from the map software in real time according to the vehicle route. Current science and technology support UAV-based wireless networks to obtain the location of all ground users in the coverage area in real time. Therefore, we think this work is feasible.

***Comment 14:*** *Why are some of the results in the reference section. The authors should fix this.*

**Response:** Theorem 1 used bisection method, which had been proved by [28] in 1967 and been widely used by a large number of studies. We think this work can be directly cited without proving it again.

***Comment 15:*** *Some key parameters are missing from the parameter table. No pathloss exponent, transmit power, clustering metrics used, etc.*

**Response:** We are really sorry for the unclear description. The clustering metrics are sum-rate and EE. The modification is complete in the section of related work..

***Comment 16:*** *In scenarios where the users are not evenly distributed in the coverage area, the size of each cluster cannot be fixed and pre-determined. Please provide some justification for this.*

**Response:** To facilitate the demonstration, we set up the user clusters can be divided equally in Fig2,3,4. But the cases with different cluster size are allowed in three user clustering algorithms. And we redescribe and explained the algorithms in the modification.

**Reviewer #2:**

***Comment：****The manuscript focus on the EE problem for UAV assisted NOMA systems. I have some concerns about the manuscript given below;
 1) There are problems with the language of the manuscript. It requires a detailed proofreadimg.*

**Response:** Thank you for your valuable comment. The manuscript has been carefully revised with red font.

*2) Why we require clustering mechanism here?*

**Response:** Thank you for your valuable comment, the illustration and modification are complete in the related work section.

*3) There are problems in the part that explains clustering algorithms.*

*i) For example in greedy algorithm the readers are directed to the Algorithm 1 and 2 at the same time.*

**Response:** We are sorry for the work didn’t add positioning to Algorithm 1 and 2. It is reasonable that the Algorithms belong to table in typesetting of Latex and appear below error:

Thus, we cancel positioning of Algorithms.

*ii) Moreover, the users are not classified as Class A and B in this algorithm, although it is written as " After users were sorted, all users were divided into two kinds of class-A and class-B."*
 **Response**: Thank you for your valuable comment, the modification is complete.

*iii) Moreover, in the text, the authors say that "The clustering result is showed in Fig 2, which rectangular blocks of the same color represent the same cluster." but there is no color in the figure.
iv) The Figures 2,3 and 4 require much more explanations. In Figures 3 and 4, why the U3 and U4 are eliminated?*
  **Response**: Thank you for your valuable comment, the modification is complete. The reason of this problem is that graphic data isn’t updated.

*iv) In Algorithm 2, why only class B clusters are given? We can not see the class A in the pseudo code.*

**Response**: Thank you for your valuable comment, the modification of Figure 3 and Figure 4 is complete.

*v) Algorithm 1, 2 and 3 must be rewritten and must be explained better.*

**Response**: Thank you for your valuable comment, the modification of Algorithm 1, 2 and 3 is complete, which referred to references [31]:

*vi) In Figure 5b,* *the trend of the EE is normal? In the EE models, it is expected that EE is saturated after a Pmax value. This part can also be explaned in detail.*

**Response**: Thank you for your valuable comment, we have revised the experiment carefully.

*vii) The EE values seem to low. They are all in bits/Joule. Generally these values are given in Mbits/Joule. Is there any problem for these values?*

**Response**: Thank you for your valuable comment, we revalidated and refined the parameters and measuring units.

*viii) The complexity of the algorithms must also be given. Although, the authors study on the number of iterations, it is not a measure for the complexity of the system. If these complexity issues are given, it will be more fair to compare non-clustering OMA and cluster-based NOMA algorithms. The EE advantage of NOMA is also slightly different compared to OMA. If the complexity of NOMA is higher, this EE gain may be compansated.*

**Response**: Thank you for your valuable comment, the computational complexity of proposed algorithms have been added in our revised manuscript.

*ix) Please check all equations. For example in Eq. (12), I think it will be R_total (q(t)) instead of R_totalq(t). Also in Eqn. (13) R_totalq(l) is given in the objective function but it is defined as Rtotal [l] in the subjective function. All the notations must be consistent.*

**Response**: We are really sorry for the grammatical errors, similar errors have been checked in our revised manuscript.

**Reviewer #3:**

*In this paper, the authors have proposed a NOMA-UAV based energy efficiency scheme and solved the problem based on three clustering algorithms. There are some important issues that should be considered.*

***Comment 1:*** *The writing of the article has many mistakes and problems in content continuity, fluency and explanations and, there are many grammatical errors and wrong statements. Especially in numerical results section. Thus all parts should be rewritten.*
  **Response:** Thank you for your valuable comment. The manuscript has been carefully revised with red font.

***Comment 2:*** *The novelty of the paper is low. Only, in the problem solution, authors use the combination of two spectrum allocations scheme named hybrid clustering.* **Response:** Thank you for your valuable comment, we have take into account the comment carefully and reorganize the contributions of this paper:

(1) This paper considers a UAV-assisted communication system where a single UAV is utilized as ABS connecting multiple ground user with NOMA. A theoretical model of propulsion energy consumption varies with flying velocity is builded, based on which, a corresponding non-convex problem with EE is proposed.
(2) To obtain the optimal solution, we transform the original problem into a convex optimization problem. A maximize EE algorithm including resource allocation scheme exploiting Dinkelbach method is proposed to solve the optimization problem. Simulation results show that the proposed algorithm is superior to other benchmarks.
(3) To verify the validity of the algorithm, three user clustering algorithms are designed with different ways of grouping, where including greedy clustering, suboptimal clustering and hybrid clustering algorithms. The simulation results indicate that different user clustering algorithms have different influence on the EE of UAV system and the performance of hybrid clustering algorithm outperforms the other two algorithms.

***Comment 3:*** *In SIC system, to interference cancelation, assigned power to the user should be allocated according to the channels gain, thus,* *this constraint should be applied in the problem, please refer to the reference 31.*
 **Response:** Thank you for your valuable comment, the manuscript has been applied the constraint.

***Comment 4:*** *In equation 12, C3 can cover C2.* **Response:** Thank you for your valuable comment, we revised the constraint by careful consideration .

***Comment 5:*** *In line 180, authors assume no-intra interference among user. Thus, no need to use NOMA.*

**Response**: We are really sorry for the theoretical and obvious errors, we revised the description by careful consideration.

***Comment 6:*** *In section 4.1.1, what is the meaning of* *the paragraph 2?*
**Response**: We are really sorry for the problem, the section has been rewritten.

***Comment 7:*** *The explanations of the Fig 6 are not clear and reviewer thinks Fig.6 has no information.*

**Response**: Thank you for your valuable comment. The manuscript has been carefully revised with red font.

***Comment 8:*** *In* *Fig 5a, sum rate is around 815 bits for all NOMA scheme and there are no superior differences between them.*

**Response**: We are really sorry for the errors, the experiment has been revalidated and the figure has been redrawn by modifying certain parameters in Fig 7a.
