# Supplementary material for: Energy-efficient UAV communication: A NOMA scheme with resource allocation and trajectory optimization
Source: PLoS One. 2024 Apr 16;19(4):e0301819. doi: 10.1371/journal.pone.0301819 (PMC11020866; doi:10.1371/journal.pone.0301819)
Supplement: Attachment — Submitted filename: Response to Reviewers 2.docx [file pone.0301819.s003.docx]

**Dear Editor and Reviewers:**

On behalf of my co-authors, we are very grateful to you for giving us the last opportunity to revise our manuscript. We appreciate you very much for your positive and constructive comments and suggestions on our manuscript entitled “A NOMA Scheme with Resource Allocation and Trajectory Optimization” (PONE-D-23-21957)

We have studied the comments of all Reviewers carefully and tried our best to revise our manuscript according to the comments. The following are the responses and revisions. We have made in response to the questions and suggestions on an item-by-item basis. Thanks again to the hard work of the editor and reviewers.

**Reviewer #1:**

***Review 1:*** *The authors have not been able to address several comments highlighted in the first review. The authors should address* *Comment 8, where they do not provide the control overhead for the proposed algorithm.*

**Response:** We are really sorry for the unclear responses. Next, we’ll reword the comments for the first review.

*Comment 8: The authors will do well to provide the control overhead of the proposed approach with respect to the baselines used in the evaluation.*

Response: We are really sorry that we did not explain the response clearly. We are regarded control overhead as the computational complexity of proposed algorithms. The related description is referred on Page 15 line 387-396:

According to total number of users is $U$, the computational complexity of greedy clustering algorithm is denoted as $O(U^{3})$ where refers to bubble sort’ complexity $O(U^{2})$ and searching clusters’ complexity $O(U)$. The complexity of suboptimal clustering algorithm and hybrid clustering algorithm are set as $O(U^{2}log_{2}U)$ and $O(U^{3}log_{2}\frac{U}{2})$, respectively. In addition, the computational complexity of two iterative algorithms are denoted as $O(OE)$, where $O$ is the iteration number of Algorithm 4 and bounded by $O_{\max}$, $E=IO(U^{3})$ is the computational complexity of Algorithm 5 due to the complexity of (21) is $O(U^{3})$and $I$ is the iteration number bounded by $I_{\max}$.

*Comment 16: In scenarios where the users are not evenly distributed in the coverage area,* *the size of each cluster cannot be fixed and pre-determined. Please provide some justification for this.*

Response: We are really sorry that we did not explain the response clearly. The signals are superimposed transmission in NOMA. To facilitate the demonstration, we consider the simplest scene that each cluster has minimum number of users (two users) in experiments. We set up the user clusters can be divided equally in Fig2,3,4 on Page 10-12. But the cases with different cluster size are allowed in three user clustering algorithms. In scenarios where the users are not evenly distributed, we still can sort ground users according to the channel gain differences complied with the basic principles of three clustering algorithms. Thus, the difference between the two cases mentioned above can be ignored.

***Review 2:*** *From Comment 11, the authors have set the height increment for each flight to 50m. How is this possible and under what consideration? This is highly impractical as it may jeopardise the findings in the results. The authors may have to reconsider their choice of 50m as this may affect the validity of the results.*

*Comment 11: How did the authors arrive at the chosen altitude for the fixed-winged UAVs?*

**Response:** We are really sorry for our unclear expression. In the work, we consider uniform velocity under ideal conditions, which means that the height of the UAV's upward flight per second is consistent. Therefore, we set a height increment for each flight slot. The height increment is 50m, which is determined by the velocity of the flight. We select 50m as an experimental standard, which won’t impact on the final experimental results.

 ***Review 3:*** *The authors are answering a different question from what was being asked in Comment 14 and 15.*

**Response:** We are really sorry for our unclear expression. Next, we’ll reword the comments for the first review.

*Comment 14: Why are some of the results in the reference section. The authors should fix this.*

Response: Thank you for your valuable comment, we deeply realize the problem and have revised it. We have added the proof of Theorem 1 according to Dinkelbach method on Page 13 line 357-362: From equation (14), we have equation $\max_{\text{p}^{*}\text{,q}[l]^{*}} \bar{R}_{total}^{*}\text{(p}^{*}\text{,q}[l]^{*})-\eta\bar{E}^{*}\text{(p}^{*}\text{,q}[l]^{*})$ based on Dinkelbach method. If $Q^{*}(\text{p}^{*},\text{q}\left[l\right]^{*})$ is used to denote $\bar{R}_{total}^{*}\text{(p}^{*}\text{,q}[l]^{*})-\eta^{*}\bar{E}^{*}\text{(p}^{*}\text{,q}[l]^{*})$, we can obtain $\eta=\eta^{*}\Leftrightarrow M(\text{p},\text{q}\left[l\right],\eta)=Q^{*}(\text{p}^{*},\text{q}\left[l\right]^{*},\eta^{*})=0$, which has been proved in formal literatures [28]. $M(\text{p},\text{q}\left[l\right],\eta)$ denotes the optimal value of $\max_{\text{p}^{*}\text{,q}[l]^{*}} Q^{*}(\text{p}^{*},\text{q}\left[l\right]^{*})$. For $\eta$, $M(\text{p},\text{q}\left[l\right],\eta)$ is a monotonically increasing convex function. Thus, the problem (P2) is written as equation (16).

In addition, we also add the explanation of Algorithm 5 on Page 15 line 382-385: From $f\text{(p}^{I})-g\text{(p}^{I})\leq f\text{(p}^{I+1})-g\text{(p}^{I})-\nabla g\text{(p}^{I})(\text{p}^{I+1}-\text{p}^{I})$, we obtain $g\text{(p})\leq g\text{(p}^{I})-\nabla g\text{(p}^{I})(\text{p}-\text{p}^{I})$ at any $\text{p}$. Then, we derive that $f\text{(p}^{I})-g\text{(p}^{I})\leq f\text{(p}^{I+1})-g\text{(p}^{I+1})$. Thus, the objective value $\Psi(\text{p})$ is improved in each iteration and the power allocation sequence $\{\text{p}^{I}\}$ is converged by Cauchy theorem.

*Comment 15: Some key parameters are missing from* *the parameter table. No pathloss exponent,* *transmit power, clustering metrics used, etc.*

Response: We are really sorry for the unclear description. (i) The pathloss exponent is transformed into the denominator of equation (5), which is shown on Page 6 line 197. (ii) The maximum transmission power P_max_ of UAV is considered as the parameter of transmit power, which is shown on Page 6 Table 2. (iii) The algorithms consider sum rate and EE as the performance indicators on Page 9 line 267, which are also our objectives for this work. We think it doesn’t need to appear in the parameter table.

**Reviewer #2:**

 ***Review:*** *It is difficult to follow if the authors addressed all questions of the reviewers. The revised parts in the manuscripts must be emphasized in the answers document. For example, the explanation has been added* *on Page XX line xxx.*

**Response:** We are really sorry for the issue and will reword the responses according to the pattern.

*Comment：2) Why we require clustering mechanism here?*

Response: Thank you for your valuable comment. UAV are energy-constrained and ground users face the challenge of interference from others who share the same frequency spectrum with them, which influences the EE of the system. Thus, we focus on dividing users into multiple clusters to improve EE in NOMA. Each cluster contains a certain number of users and scheduling resources, which ensures that there is no interference among users. The illustration and modification are completed in the related work section in the last paragraph on Page 3.

*3) There are problems in the part that explains clustering algorithms.*

*ii) Moreover, the users are not classified as Class A and B in this algorithm, although it is written as " After users were sorted, all users were divided into two kinds of class-A and class-B."*

*iv) In Algorithm 2,* *why only class B clusters are given? We can not see the class A in the pseudo code.* Response: Thank you for your valuable comment. We have modified Fig 3 and 4 to clearly show how class-A and class-B are classified on Page 11 and 12. The class-A and class-B are followed by inter-group clustering in such a way that middle users are primely accommodated in [31] on Page 10 line 297.

*iv) The Figures 2,3 and 4 require much more explanations. In Figures 3 and 4, why the U3 and U4 are eliminated?*

Response: Thank you for your valuable comment. The explanations about Figures 2,3 and 4 are revised in chapter 4 on Page 8-12. In addition, the graphic data of Figures 3 and 4 are updated on Page 11 and 12.

*viii) The complexity of the algorithms must also be given. Although, the authors study on the number of iterations, it is not a measure for the complexity of the system. If these complexity issues are given, it will be more fair to compare non-clustering OMA and cluster-based NOMA algorithms. The EE advantage of NOMA is also slightly different compared to OMA. If the complexity of NOMA is higher, this EE gain may be compansated.*

Response: Thank you for your valuable comment. According to total number of users is $U$, the computational complexity of greedy clustering algorithm is denoted as $O(U^{3})$ which refers to bubble sort’ complexity $O(U^{2})$ and searching clusters’ complexity $O(U)$. The complexity of suboptimal clustering algorithm and hybrid clustering algorithm are set as $O(U^{2}log_{2}U)$ and $O(U^{3}log_{2}\frac{U}{2})$, respectively. In addition, the computational complexity of two iterative algorithms are denoted as $O(OE)$, where $O$ is the iteration number of Algorithm 4 and bounded by $O_{\max}$, $E=IO(U^{3})$ is the computational complexity of Algorithm 5 due to the complexity of (21) is $O(U^{3})$and $I$ is the iteration number bounded by $I_{\max}$. Different from non-clustering OMA, the complexity of NOMA is obviously higher. But the performance of proposed algorithms is improved by more than 99.6%, 104%, and 111% respectively on Page 16 line 424. The computational complexity of proposed algorithms have been added in our revised manuscript on Page 15 line 387-395.

**Reviewer #3:**

***Review 1:*** *Although the authors have improved the article and added many explanations unfortunately the article still is not fluent and there are lots of grammatical mistake especially in new added parts.*

**Response:** We are really sorry for the grammatical mistakes. We have carefully revised the manuscript with red font again. Thank you for your valuable comment.

***Review 2:*** *As the novelty of the work is low, according to table 1. It is necessary that the performance of the work is compared with those in the some references such as 24 and 26 .*

**Response:** Thank you for your valuable comment very much, we have revised the manuscript on Page 4 line 131-133.

***Review 3:*** *What is 'm' in the C4 equation?*

**Response:** We are really sorry for the error. The letter ‘m’ should be written as ‘n’ and the modification is complete on Page 13 line 365 for equation (16), Page 14 line 378 for equation (21).

Yours truly

Shuang Zhang
