# Supplementary material for: Energy-efficient UAV communication: A NOMA scheme with resource allocation and trajectory optimization
Source: PLoS One. 2024 Apr 16;19(4):e0301819. doi: 10.1371/journal.pone.0301819 (PMC11020866; doi:10.1371/journal.pone.0301819)
Supplement: Attachment — Submitted filename: Response to Reviewers 3.docx [file pone.0301819.s004.docx]

**Dear Editor and Reviewers:**

On behalf of my coauthors, I am grateful to you for giving us this opportunity to revise our manuscript. We appreciate your positive and constructive comments and suggestions on our manuscript titled “A NOMA Scheme with Resource Allocation and Trajectory Optimization” (PONE-D-23-21957).

We have studied the comments of all the Reviewers carefully and tried our best to revise our manuscript accordingly. The following summarizes the responses and revisions. We have responded to the questions and suggestions on an item-by-item basis. Thank you again for the hard work of the editor and reviewers.

**Reviewer #1:**

***Review:*** T*he authors have addressed all my comments. I believe the paper is in a much better form. Hence, I recommend that it be accepted. Thanks.*

**Response:** Thank you very much for your approval of the manuscript. We will strive to further improve the paper.

**Reviewer #2:**

***This Review:*** *The authors did not answer all questions point by point. They seem to prefer select some of them to answer, that is not a good way.*

**Response:** We apologize for this issue because of our unclear understanding. Next, we will reply to the previous questions point by point.

 ***The Second Review:*** *It is difficult to follow if the authors addressed all questions of the reviewers. The revised parts* *in the manuscripts must be emphasized in the answers document. For example, the explanation has been added on Page XX line xxx.*

**Response:** We apologize for this issue and have reworded the responses accordingly.

***The First Review:*** *The manuscript focus on the EE problem for UAV assisted NOMA systems. I have some concerns about the manuscript given below;
 Comment 1：There are problems with the language of the manuscript. It requires a detailed proofreadimg.*

Response: Thank you for your valuable comment. We have paid close attention to this issue. The manuscript has been carefully revised, and the changes are shown in red font.

*Comment 2：Why we require clustering mechanism here?*

Response: Thank you for your valuable comment. UAVs are energy-constrained, and ground users face the challenge of interference from others who share the same frequency spectrum, which influences the EE of the system. Thus, we focus on dividing users into multiple clusters to improve EE in NOMA. Each cluster contains a certain number of users and scheduling resources, which ensures that there is no interference among users. The illustration and modifications have been added to the related work section in the last paragraph on Page 3.

*Comment 3：There are problems in the part that explains clustering algorithms.*

*i) For example in greedy algorithm the readers are directed to the Algorithm 1 and 2 at the same time.*

Response: We apologize that this work did not add positioning to Algorithms 1 and 2. It is reasonable that the algorithms belong to the table in the typesetting of Latex and appear as follows:

Thus, we cancel the positioning of the algorithms.

*ii) Moreover, the users are not classified as Class A and B in this algorithm, although it is written as " After users were sorted, all users were divided into two kinds of class-A and class-B."* Response: Thank you for your valuable comment. We have modified Figs 3 and 4 to clearly show how class-A and class-B are classified on Page 10 and 12. Class-A and class-B are followed by intergroup clustering such that middle users are primely accommodated in [31] on Page 10 line 278.

*iii) Moreover, in the text, the authors say that "The clustering result is showed in Fig 2, which rectangular blocks of the same color represent the same cluster." but there is no color in the figure.*
 Response: Thank you for your valuable comment. The modifications of Figs 2, 3 and 4 have been completed on Pages 9-12. The reason for this problem is that the graphic data are not updated, and the work is ignored.

*iv) In Algorithm 2, why only class B clusters are given? We can not see the class A in the pseudo code.*

Response: In Algorithm 2, B_1_-B_K_ shows the conditions of the user cluster, which are not class-B clusters. We compared the names of the clusters in Algorithms 1, 2 and 3 in the next figures. In addition, we have modified Figs 3 and 4 to clearly show how class-A and class-B are classified on Pages 9 and 11.

Figure 1. Algorithm 2

Figure 2. Algorithm 1

Figure 3. Algorithm 3

*v) Algorithm 1, 2 and 3 must be rewritten and must be explained better.*

Response: Thank you for your valuable comment. The modifications to Algorithms 1, 2 and 3 have been made carefully; refer to reference [31]:

*vi)* *In Figure 5b, the trend of the EE is normal? In the EE models, it is expected that EE is saturated after a Pmax value. This part can also be explaned in detail.*

Response: Thank you for your valuable comment. We have revised the experiment and figure carefully for Fig 5. The relevant description has been revised in the last paragraph on Page 16.

*vii) The EE values seem to low. They are all in bits/Joule. Generally these values are given in Mbits/Joule. Is there any problem for these values?*

Response: Thank you for your valuable comment. We have revalidated and refined the parameters and measuring units in Section 5.

*viii) The complexity of the algorithms must also be given. Although, the authors study on the number of iterations, it is not a measure for the complexity of the system. If these complexity issues are given, it will be more fair to compare non-clustering OMA and cluster-based NOMA algorithms. The EE advantage of NOMA is also slightly different compared to OMA. If the complexity of NOMA is higher, this EE gain may be compansated.*

Response: Thank you for your valuable comment. According to the total number of users $U$, the computational complexity of the greedy clustering algorithm is denoted as $O(U^{3})$, which refers to the complexity of the bubble sort $O(U^{2})$ and the complexity of the searching clusters $O(U)$. The complexities of the suboptimal clustering algorithm and hybrid clustering algorithm are set as $O(U^{2}log_{2}U)$ and $O(U^{3}log_{2}\frac{U}{2})$, respectively. In addition, the computational complexity of the two iterative algorithms is denoted as $O(OE)$, where $O$ is the iteration number of Algorithm 4 and bounded by $O_{\max}$, $E=IO(U^{3})$ is the computational complexity of Algorithm 5 because the complexity of (21) is $O(U^{3})$and $I$ is the iteration number bounded by $I_{\max}$. In contrast to that of nonclustering OMA, the complexity of NOMA is obviously greater. However, the performance of the proposed algorithms is improved by more than 99.6%, 104%, and 111%, respectively, on Page 15 line 408. The computational complexity of the proposed algorithms has been added to our revised manuscript on Page 14 lines 362-378.

*ix) Please check all equations. For example in Eq. (12), I think it will be R_total (q(t)) instead of R_totalq(t). Also in Eqn. (13) R_totalq(l) is given in the objective function but it is defined as Rtotal [l] in the subjective function. All the notations must be consistent.*

Response: We apologize for the grammatical errors. Similar errors have been addressed in our revised manuscript on Page 7, Equations (11) and (12); Page 8, Equation (13).

**Reviewer #3:**

***Review:*** *Authors have revised the article and almost answered the questions but there are some problems in the structure and statements of the article. For example:
 1- Last statement of the abstract.*

Response: Thank you for your valuable comment. We have revised the manuscript with red on Page 1 in Abstract.

*2- Statements in line 9-10 , in line 34-35 and in line 42-43 in introduction section.*

Response: Thank you for your valuable comment. We have revised the manuscript with red on Page 2 and 3.

3- *Furthermore, there are many mistakes in other parts of the article that should be revised.*

Response: We apologize for the grammatical mistakes. We have carefully revised the manuscript, and the changes are shown in red. Thank you very much for the valuable comment.
