# Supplementary material for: Energy-efficient UAV communication: A NOMA scheme with resource allocation and trajectory optimization
Source: PLoS One. 2024 Apr 16;19(4):e0301819. doi: 10.1371/journal.pone.0301819 (PMC11020866; doi:10.1371/journal.pone.0301819)
Supplement: Attachment — Submitted filename: Response to Reviewers'.docx [file pone.0301819.s005.docx]

**Reviewer #2:**

***The Third Review:*** *The authors did not answer all questions point by point. They seem to prefer select some of them to answer, that is not a good way.*

**Response:** We apologize for this issue because of our unclear understanding. Next, we will reply to the previous questions point by point.

***The Second Review:*** *It is difficult to follow if the authors addressed all questions of the reviewers. The revised parts* *in the manuscripts must be emphasized in the answers document. For example, the explanation has been added on Page XX line xxx.*

**Response:** We apologize for this issue and have reworded the responses accordingly.

***The First Review:*** *The manuscript focus on the EE problem for UAV assisted NOMA systems. I have some concerns about the manuscript given below;
 Comment 1：**There are problems with the language of the manuscript. It requires a detailed proofreadimg.*

Response: Thank you for your valuable comment. We have paid close attention to this issue. Currently, the language of the manuscript has undergone several rounds of revisions and has been polished by a language editing service. Thank you very much for your comment again.

*Comment 2：Why we require clustering mechanism here?*

Response: Thank you for your valuable comment. NOMA technology enables multiple users to transmit signals on the same resource block simultaneously. Therefore, the clustering mechanism based on the user channel gain differences can be a necessary step in the operation of NOMA. The algorithms of clustering mechanism determine the quality of signal transmission. Therefore, this paper considers the user clustering algorithms based on NOMA. The working principle of NOMA is described on **Page 2 line 24-26.**

*Comment 3：There are problems in the part that explains clustering algorithms.*

*i) For example in greedy algorithm the readers are directed to the Algorithm 1 and 2 at the same time.*

Response: We apologize that this work did not add positioning to Algorithms 1 and 2. It is reasonable that the algorithms belong to the table in the typesetting of Latex and appear as follows:

Thus, we cancel the positioning of the algorithms.

*ii) Moreover, the users are not classified as Class A and B in this algorithm, although it is written as " After users were sorted, all users were divided into two kinds of class-A and class-B."* Response: Thank you for your valuable comment.

1) The illustration and modifications have been added to the related work section on **Page 9 line 273-277:** Overall, users are classified into class A and class B on the basis of channel gains, followed by intergroup clustering in such a way that middle users are primely accommodated. The number of users in class A is denoted as M, which is provided with a much smaller channel gain than that of users in class B; this number is denoted as U-M.

2) We have modified **Figs 3 and 4 with red** to clearly show how class-A and class-B are classified on **Page 10 and 12**. Class-A and class-B are followed by intergroup clustering such that middle users are primely accommodated on **Page 10 line 281-283**.

*iii)* *Moreover, in the text, the authors say that "The clustering result is showed in Fig 2, which rectangular blocks of the same color represent the same cluster." but there is no color in the figure.*
 Response: Thank you for your valuable comment. The modifications of **Figs 2, 3 and 4** have been completed with red **on Pages 9-12**. The reason for this problem is that the graphic data are not updated, and the work is ignored.

*iv) In Algorithm 2, why only class B clusters are given? We can not see the class A in the pseudo code.*

Response: Thank you for your valuable comment. In Algorithm 2, B_1_-B_K_ shows the conditions of the user cluster, which are not class-B clusters on **the top with Pages 10**. We compared the names of the clusters in Algorithms 1, 2 and 3 in the next figures. In addition, we have modified **Figs 3, 4 with red** to clearly show how class-A and class-B are classified.

Figure 1. Algorithm 2

In Algorithm 1, A_1_-A_K_ shows the conditions of the user cluster on **the top with** **Page 9**, which are also not class-A clusters.

Figure 2. Algorithm 1

In Algorithm 3, C_1_-C_K_ shows the conditions of the user cluster on **Page 11 line 302-303**.

Figure 3. Algorithm 3

In addition, The class A and class B clusters are allocated according to the rules:

*v) Algorithm 1, 2 and 3 must be rewritten and must be explained better.*

Response: Thank you for your valuable comment. The descriptions of **Algorithms 1, 2 and 3** have been rewritten with red carefully on **the top with** **Page 9 and 10; Page 11 line 302-303**; refer to reference [31]:

*vi)* *In the EE models, it is expected that EE* *is saturated after a Pmax valuein Figure 5b. This part can also be explaned in detail.*

Response: Thank you for your valuable comment. We have revised the experiment and figure carefully for Fig 5. The supplementary description has been revised on **Page 15 line 391-398:** In addition, as shown in Fig 5(b), the variation in EE with alternating iterations is explored. After one or two iterations, the EE of Algorithm 4 converges to the approximately global optimizal solution by calculating the space convergence feature and optimality of the proposed algorithm. Clearly, the superiority of the NOMA scheme is revealed in that it produces greater performance gain when users are equipped with highly different channel conditions. The simulations indicate that the performance of EE with the proposed algorithms is improved by more than 99.6%, 104%, and 111%, respectively.

*vii) The EE values seem to low. They are all in bits/Joule. Generally these values are given in Mbits/Joule. Is there any problem for these values?*

Response: Thank you for your valuable comment. The low value of EE is due to that we neglected bandwidth in the first work. The bandwidth has now been added EE and compared with values from other literatures. The results show that the current EE value is correct. We have revalidated and refined the parameters and measuring units with **Figs 5 on Page 15; Figs 7 on Page 17**.

*viii) The complexity of the algorithms must also be given. Although, the authors study on the number of iterations, it is not a measure for the complexity of the system. If these complexity issues are given, it will be more fair to compare non-clustering OMA and cluster-based NOMA algorithms. The EE advantage of NOMA is also slightly different compared to OMA. If the complexity of NOMA is higher, this EE gain may be compansated.*

Response: Thank you for your valuable comment. The computational complexity of the proposed algorithms has been added to our revised manuscript on **Page 14 lines 361-370:** According to the total number of users $U$, the computational complexity of the greedy clustering algorithm is denoted as $O(U^{3})$, which refers to the complexity of the bubble sort $O(U^{2})$ and the complexity of the searching clusters $O(U)$. The complexities of the suboptimal clustering algorithm and hybrid clustering algorithm are set as $O(U^{2}log_{2}U)$ and $O(U^{3}log_{2}\frac{U}{2})$, respectively. In addition, the computational complexity of the two iterative algorithms is denoted as $O(OE)$, where $O$ is the iteration number of Algorithm 4 and bounded by $O_{\max}$, $E=IO(U^{3})$ is the computational complexity of Algorithm 5 because the complexity of (21) is $O(U^{3})$and $I$ is the iteration number bounded by $I_{\max}$. In contrast to that of nonclustering OMA, the complexity of NOMA is obviously greater. However, the performance of the proposed algorithms is improved by more than 99.6%, 104%, and 111%, respectively, **on Page 15 line 397-398**.

*ix) Please check all equations. For example in Eq. (12), I think it will be R_total (q(t)) instead of R_totalq(t). Also in Eqn. (13) R_totalq(l) is given in the objective function but it is defined as Rtotal [l] in the subjective function. All the notations must be consistent.*

Response: We apologize for the grammatical errors. Similar errors have been addressed in our revised manuscript with red on **Page 7, Equations (11) and (12); Page 8****, Equation (13).**
